# Supplementary material for: The Effect of Temperature on the Embryo Development of Cephalopod Sepiella japonica Suggests Crosstalk between Autophagy and Apoptosis
Source: Int J Mol Sci. 2023 Oct 19;24(20):15365. doi: 10.3390/ijms242015365 (PMC10607546; doi:10.3390/ijms242015365)
Supplement: Supplementary file 1 [file ijms-24-15365-s001.zip › ijms-2653066-supplementary.pdf]

Tables

Table S1. Primers used for real-time quantitative PCR

**Genes**	**Sequences (5'-3')**	**References**
β-actin-F	GCCAGTTGCTCGTTACAG	JN564496.1
β-actin-R	GCCAACAATAGATGGGAAT	
GAPDH-F	TGGTTCCTTGGCTTTTGCT	Huo et al., 2018
GAPDH-R	GGTGGTGGTGCGGGTAGT	
Inx4-F	ACGCGCCATATAATACACGG	
Inx4-R	CGTGGTCCCTAGCGAGATAC	
LC3-F	CGCCGTTTACAGTTGCATCC	
LC3-R	GCCGTCGTCATCTTTTTCCC	
BECN1-F	GCCAACTGGAAAAATTGACC	
BECN1-R	AGAATCGGAATCCTCCTGAA	
Cas3-F	TGCAAAAACGGTTCCTGGTT	
Cas3-R	AGTCAACATAGAGGCAACGCA	
p53-F	GCCTGTATCATCAACTCCAT	
p53-R	AGAATAAGTCCAGGTTGTAG	
